# Synergistic Integration of SrFeO_3_ Nanoparticles With Exfoliated MXene (Ti_3_C_2_T*_x_*/V_2_CT*_x_*) Nanosheets for High Performance Supercapacitors

**DOI:** 10.1002/smll.74641

**Published:** 2026-08-04

**Authors:** Zulfqar Ali Sheikh, Shahzaib Ali, Sayed Zafar Abbas, Dhanasekaran Vikraman, Faisal Shahzad, Doyoung Byun, Hyun‐Seok Kim, Sajjad Hussain, Jongwan Jung

**Affiliations:** ^1^ Hybrid Materials Center (HMC) Sejong University Seoul South Korea; ^2^ Department of Nanotechnology and Advanced Materials Engineering Sejong University Seoul South Korea; ^3^ Department of Mechanical Engineering Sungkyunkwan University Suwon Gyeonggi‐do South Korea; ^4^ Division of Electronics and Electrical Engineering Dongguk University‐Seoul Seoul South Korea; ^5^ Research and Innovation Center for Graphene and 2D Materials (RIC2D) Khalifa University Abu Dhabi UAE

**Keywords:** capacitance, energy storage, faraday efficiency, heterojunction, materials science, nanoparticle, power density, pseudocapacitor, supercapacitor

## Abstract

The performance of supercapacitors is often limited by conventional electrode materials, which typically necessitate a compromise between energy density, power density, and cycling stability. While two‐dimensional MXenes offer high conductivity and surface area, their practical application is hindered by restacking and oxidative degradation. This study introduces a novel heterostructured composite designed to overcome these limitations. For the first time, we fabricate a porous, exfoliated network by integrating zero‐dimensional SrFeO_3_ nanoparticles with Ti_3_C_2_T*_x_* and V_2_CT_x_ MXenes via a straightforward mechanical mixing process. In this architecture, the SrFeO_3_ nanoparticles fulfill a dual role: they inhibit MXene restacking and contribute significant pseudocapacitance via Faradaic reactions. The synergistic coupling between the conductive MXene scaffolds and the redox‐active nanoparticles yields exceptional electrochemical performance. The optimized Ti_3_C_2_T*_x_* @SrFeO_3_ and V_2_C@SrFeO_3_ electrodes achieve specific capacitances of 752 and 972 F g^−^^1^, respectively, at 1 A g^−^^1^, alongside excellent rate capability. Asymmetric supercapacitor devices assembled with these composites deliver high energy densities of up to 52.91 Wh kg^−^^1^ and exhibit outstanding long‐term stability, retaining over 93.9% of their initial capacitance after 5000 cycles. This work establishes the MXene/SrFeO_3_ heterostructure as a promising platform for high performance energy storage.

## Graphical abstract

A facile mechanical mixing, probe‐sonication, and annealing strategy was employed to intercalate SrFeO3 nanoparticles between Ti_3_C_2_T*_x_* and V_2_CT*_x_* MXene sheets, constructing robust heterostructures for advanced supercapacitors. The inserted perovskite suppresses MXene restacking while contributing additional Faradaic redox activity. The optimized hybrid delivers a high specific capacitance of 972 F g^−^^1^, an ASC energy density of 52.91 Wh kg^−^^1^, and 93.9% capacitance retention after 5000 cycles.

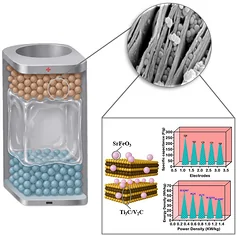

## 1 Introduction

The growing energy crisis and environmental pollution urgently demand sustainable energy solutions. Supercapacitors, with high power density, fast charging, and long cycle life, outperform conventional batteries and fuel cells for energy storage [1, 2]. Key advancements in solar energy conversion (photovoltaics, water splitting) and electrochemical storage (batteries, supercapacitors) are critical [3, 4, 5]. The development of high performance supercapacitors is critically dependent on finding electrode materials that are not only low‐cost and abundant but also possess high capacitance, energy density, power density, and long‐term stability. In this pursuit, the MXene family, particularly Ti_3_C_2_T*_x_* and V_2_CT*_x_*, has emerged as a highly promising class of materials [6, 7]. Their suitability stems from a unique combination of properties, including intrinsic metallic conductivity, hydrophilic surfaces that facilitate electrolyte access, and a layered two‐dimensional structure that offers a high surface area for ion adsorption [8, 9]. Within this family, Ti_3_C_2_T*_x_* and V_2_CT*_x_* serve distinct roles. Ti_3_C_2_T*_x_* is a versatile benchmark material, valued for its exceptional electrical conductivity and a mixed charge storage mechanism that combines electric double‐layer capacitance with surface redox pseudocapacitance [10, 11]. This results in devices with very high power density and robust cycle life. V_2_CT*_x_* MXene is a prominent candidate for high‐energy‐density supercapacitors, leveraging the multi‐electron redox chemistry of vanadium to deliver high pseudocapacitance and a wide voltage window [12, 13]. However, its practical application is constrained by intrinsic challenges: lower electronic conductivity, susceptibility to oxidation, and the propensity for nanosheet restacking driven by van der Waals forces and hydrogen bonding. This restacking diminishes the electrochemically active surface area and can accelerate oxidative degradation, ultimately impairing rate capability and long‐term cyclability [14]. A common strategy to mitigate this and improve mechanical flexibility is to form composite structures with materials like transition metal dichalcogenides (TMDs) [15], polymers [16, 17], carbon based nanoparticles [18, 19], or metal oxides [20, 21]. Integrating perovskite oxides presents a particularly advantageous solution. Perovskites (ABO_3_) possess a flexible, cubic structure where the *B*‐site transition metal cation (e.g., Fe, Co) is octahedrally coordinated by oxygen (BO_6_), governing their electrochemical activity [22, 23]. Their properties, including high ionic/electronic conductivity and anion‐intercalation charge storage, can be tuned via *A*‐ and *B*‐site cation substitution. Iron‐based perovskites like SrFeO_3_ are especially promising due to iron's natural abundance, variable oxidation states, and the material's high stability and low cost [24, 25]. As nanoparticles, SrFeO_3_ acts as a physical spacer, inhibiting MXene restacking and providing a protective barrier against oxidation, thereby preserving a high interfacial area for electrolyte access. Furthermore, a synergistic effect enhances performance: the SrFeO_3_ nanoparticles contribute substantial pseudocapacitance through Faradaic redox reactions, which are efficiently facilitated by the highly conductive MXene matrix. This partnership results in a composite with a specific capacitance exceeding the sum of its individual components, while also improving the electrode's mechanical robustness. Therefore, constructing MXene/SrFeO_3_ heterostructures is a strategic approach to simultaneously overcome the inherent limitations of MXenes and unlock superior, synergistic electrochemical properties for advanced energy storage.

Herein, we demonstrate a novel architecture for supercapacitor electrodes, constructed through the straightforward mechanical mixing of two‐dimensional Ti_3_C_2_T*_x_* and V_2_CT*_x_* MXenes with 0D SrFeO_3_ nanoparticles. This process yields a porous, heterostructured network that leverages profound synergistic effects. The resulting Ti_3_C_2_T*_x_* @SrFeO_3_ and V_2_CT*_x_* @SrFeO_3_ electrodes exhibit ultrahigh specific capacitances of 1245 and 1443 F g^−^^1^, respectively, at 1 A g^−^^1^. They also display exceptional rate performance, retaining 91.1% of their initial capacitance at high current densities. When integrated into asymmetric supercapacitor (ASC) devices, the configurations achieve remarkable energy densities of 44.3 and 47.0 Wh kg^−^^1^ at power densities of 496 and 527 W kg^−^^1^, respectively. Furthermore, these devices demonstrate outstanding long‐term cycling stability, retaining 85.3% and 90.1% of their initial capacitance after 5000 cycles. This performance stems from a synergistic interplay within the composite: the MXenes provide a highly conductive scaffold for rapid electron transport, while the SrFeO_3_ nanoparticles contribute significant Faradaic pseudocapacitance and simultaneously act as spacers to mitigate MXene restacking. This simple, scalable mixing route effectively creates a hierarchical structure that facilitates ion accessibility and combines high‐energy storage with high‐power delivery.

## 2 Experimental Detail

### 2.1 Synthesis of Ti_3_C_2_T*_x_* and V_2_CT*_x_* MXenes

The synthesis of MXene was carried out by selectively etching Al from Ti_3_AlC_2_ MAX (400 mesh) using an in‐situ HF etchant prepared from HCl and LiF. Briefly, 1.6 g of LiF was dissolved in 20 mL of 9 M HCl under stirring for 10 min. Subsequently, 1 g of Ti_3_AlC_2_ was added slowly over 10 min, and the mixture was stirred in an oil bath at 36°C for 24 h under an argon atmosphere. The resulting product was washed with deionized water by repeated centrifugation/decanting cycles until the pH of the supernatant reached ∼6. The collected sediment was then shaken and recentrifuged at 4000 rpm for 5 min to 1 h over multiple cycles to promote delamination and swelling. Finally, the swelled sediment was re‐dispersed in deionized water and centrifuged at 2500 rpm for 3 min, and the dark colloidal supernatant containing Ti_3_C_2_T*_x_* was collected.

The synthesis of V_2_C MXene was carried out by selectively etching Al from V_2_AlC MAX using HF, followed by TMAOH‐assisted delamination. Briefly, 1 g of V_2_AlC was added to 20 mL of 48% HF, and the mixture was kept under continuous stirring at 48°C for 48 h. After etching, the reaction product was washed thoroughly with deionized water through repeated centrifugation/decanting cycles until the pH of the supernatant reached neutral (≈7). The neutralized etched sediment was then treated with 5 wt% TMAOH and stirred overnight to promote intercalation and delamination, followed by further washing with deionized water until a neutral pH was achieved. Finally, the dispersion was centrifuged at 2000 rpm to remove unexfoliated particles, and the dark supernatant containing delaminated V_2_C T*_x_* was collected. During this process, the Al atoms in the MAX phase are selectively removed by the following predominant reaction:

(1) $$V_{2}\mathrm{AlC}+3\mathrm{HF}\to V_{2}CT_{x}+\mathrm{Al}F_{3}+H_{2}$$

### 2.2 Synthesis of SrFeO_3_ Nanoparticles

The SrFeO_3–_δ perovskite was synthesized using a hydrothermal route. Stoichiometric quantities of strontium nitrate (Sr(NO_3_)_2_), iron(III) nitrate nonahydrate (Fe(NO_3_)_3_·9H_2_O), and urea were first dissolved in 50 mL of deionized water under vigorous stirring to form a clear precursor solution. Subsequently, a 4 M NaOH solution was added dropwise under continuous stirring until a uniform suspension was obtained, and the pH of the mixture was adjusted to above 13. The resulting suspension was transferred into a 100 mL Teflon‐lined stainless‐steel autoclave, sealed, and heated at 180°C for 15 h. After naturally cooling to room temperature, the solid product was recovered by centrifugation, and washed repeatedly with deionized water and ethanol until the supernatant reached neutral pH. The washed precipitate was dried overnight at 80°C, followed by calcination at 700°C for 2 h under an argon atmosphere to obtain the crystalline SrFeO_3–_δ phase.

### 2.3 Synthesis of Ti_3_C_2_T*_x_* @SrFeO_3_ and V_2_CT*_x_* @SrFeO_3_ Composites

**FIGURE 1:**
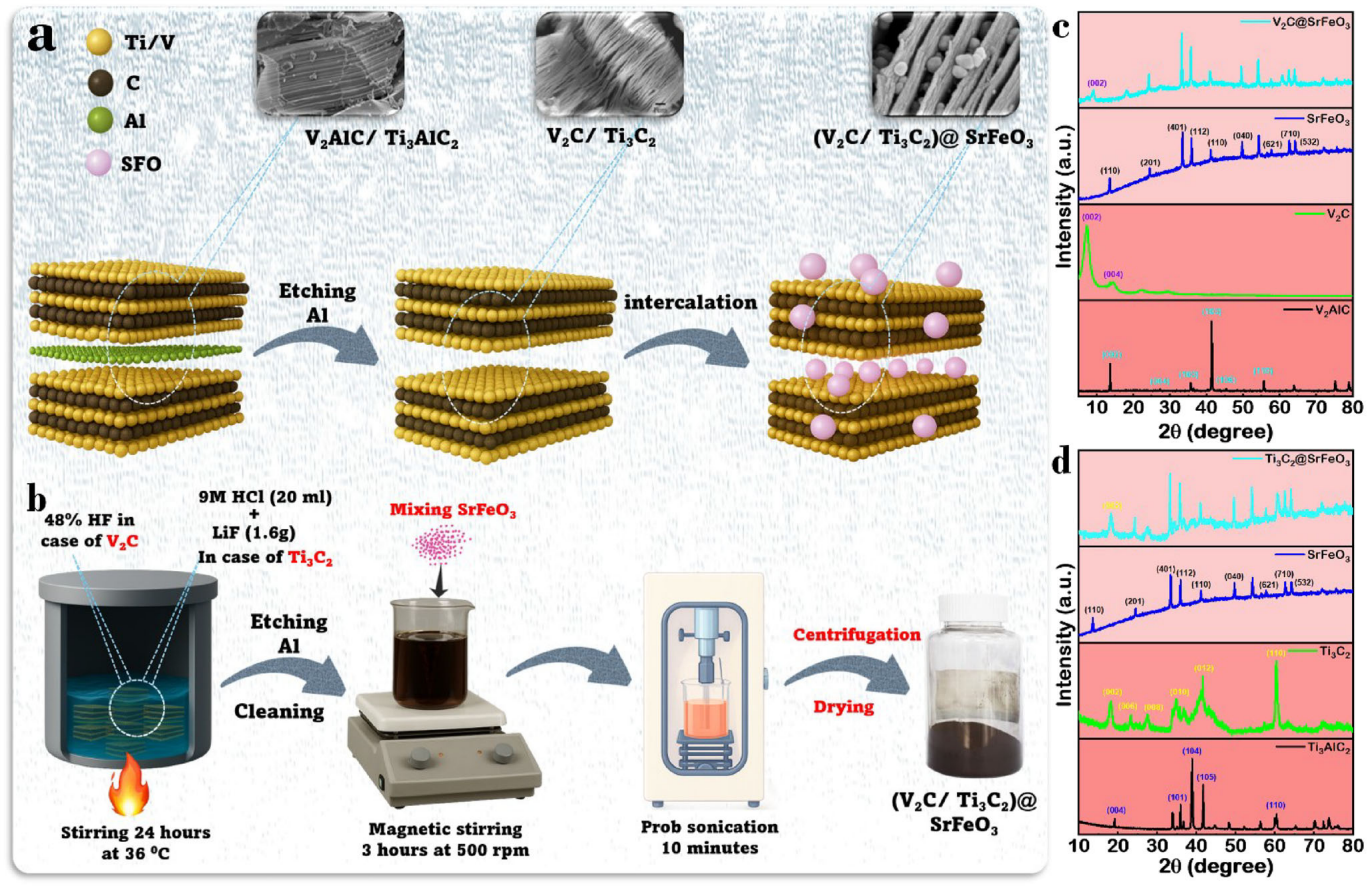
(a,b) Synthesis process and (c,d) XRD patterns of V_2_CT*_x_* @SrFeO_3_, SrFeO_3_, V_2_CT*_x_*, V_2_AlC and Ti_3_C_2_T*_x_* @SrFeO_3_, SrFeO_3_, Ti_3_C_2_T*_x_*, Ti_3_AlC_2_ hybrid materials respectively.

The MXene@SrFeO_3_ hybrid was synthesized via a sonication‐assisted intercalation strategy (Figure 1a,b). Synthesized SrFeO_3_ nanoparticles were mixed with delaminated MXene (Ti_3_C_2_T*_x_* or V_2_CT*_x_*) in a 1:1 mass ratio and magnetically stirred at 500 rpm for 3 h to ensure homogeneous dispersion. The mixture was then subjected to probe ultrasonication for 10 min, facilitating the intercalation of SrFeO_3_ nanoparticles into the MXene interlayers and suppressing restacking. The resulting suspension was centrifuged at 6000 rpm for 15 min, followed by washing and drying. The collected product was further annealed at 300°C under Ar atmosphere, enhancing interfacial contact and electrical conductivity.

The intercalated architecture provides enlarged interlayer spacing and efficient electron/ion transport pathways, enabling a synergistic combination of EDLC (MXene) and pseudocapacitance (SrFeO_3_). Consequently, the hybrid is expected to exhibit enhanced specific capacitance, rate capability, and cycling stability due to reduced charge‐transfer resistance and increased electrochemically active sites.

## 3 Results and Discussion

### 3.1 Structural Property

Figure 1a,b schematically illustrates the two‐step synthesis pathway for the Ti_3_C_2_T*_x_* @SrFeO_3_ and V_2_CT*_x_* @SrFeO_3_ composites. Initially, the MXene precursors V_2_CT*_x_* and Ti_3_C_2_T*_x_* were prepared via the selective etching of their corresponding MAX phases. Separately, SrFeO_3_ (SFO) nanoparticles were synthesized via a hydrothermal method. In the final step, the zero‐dimensional (0D) SrFeO_3_ nanoparticles were embedded between the layers of Ti_3_C_2_T*_x_* and V_2_CT*_x_* sheets to form the composites. The formation of a binary heterojunction MXene/SrFeO_3_ interface network occurs through mechanical mixing, involving physical and physicochemical processes without covalent bonding or high‐temperature annealing. During mixing, shear forces exfoliate multilayered MXene into few‐layer or single‐layer flakes, generating high‐surface‐energy basal planes and embedding SrFeO_3_ nanoparticles onto the MXene surfaces. The negatively charged MXene surfaces attract the positively charged SrFeO_3_ nanoparticles due to their mixed valence states and oxygen vacancies, promoting a uniform decoration on Ti_3_C_2_T*_x_* or V_2_CT*_x_*. This process also removes contaminants and creates undercoordinated metal sites on MXene edges, lowering the barrier for heterojunction formation. Additionally, SrFeO_3_ nanoparticles act as physical spacers, preventing the restacking of MXene sheets. Notably, the mixing process relies on vdW forces, electrostatic attraction, and mechanical interlocking without forming covalent bonds or new crystalline phases. Figure 1c,d presents the x‐ray diffraction (XRD) patterns used to confirm the phase composition and successful formation of SrFeO_3_, Ti_3_C_2_T*_x_*, V_2_CT*_x_*, and the two composites. The diffraction patterns for the pristine SrFeO_3_ and all composite samples exhibit prominent peaks that align well with the orthorhombic perovskite structure confirming the phase purity of the synthesized material. Characteristic peaks corresponding to the (1 1 0), (2 0 1), (4 0 0), (1 1 2), (0 4 0), (5 1 2), (6 2 1), (7 1 0), and (5 3 2) crystallographic planes are distinctly observed [26, 27]. The diffraction patterns of the exfoliated Ti_3_C_2_T*_x_* and V_2_CT*_x_* MXenes (Figure 1c,d) confirm successful etching of the respective Al layers from their parent MAX phases (Ti_3_C_2_T*_x_* and V_2_AlC), as evidenced by the disappearance of the dominant (104) MAX phase peak at around 39°. The patterns are dominated by the broad and intense (002) peak at a low 2θ angle (∼7–9°), which is a signature of the well‐layered, two‐dimensional structure of MXenes. A shift of this (002) peak to a lower angle in the exfoliated samples, compared to their multilayer precursors, indicates an increase in the *c*‐lattice parameter due to interlayer hydration and expansion. Critically, the XRD patterns of the Ti_3_C_2_T*_x_* @SrFeO_3_ and V_2_CT*_x_* @SrFeO_3_ composites (Figure 1c,d) provide definitive evidence of successful hybridization. The composites display all the characteristic diffraction peaks of both the crystalline SrFeO_3_ phase and their respective MXene components. However, a notable decrease in the intensity of the MXene (002) peak is observed, suggesting a partial disruption of the long‐range ordered stacking of MXene layers due to the intercalation and surface decoration by SrFeO_3_ nanoparticles. Furthermore, a slight broadening and a minor shift of the MXene (002) peak to a lower angle may be present, indicating a further expansion of the interlayer spacing caused by the incorporation of the nanoparticles. The absence of any new, extraneous peaks confirms that the integration is a physical mixture and/or a heterostructural coupling without the formation of undesirable crystalline by‐products.

**FIGURE 2:**
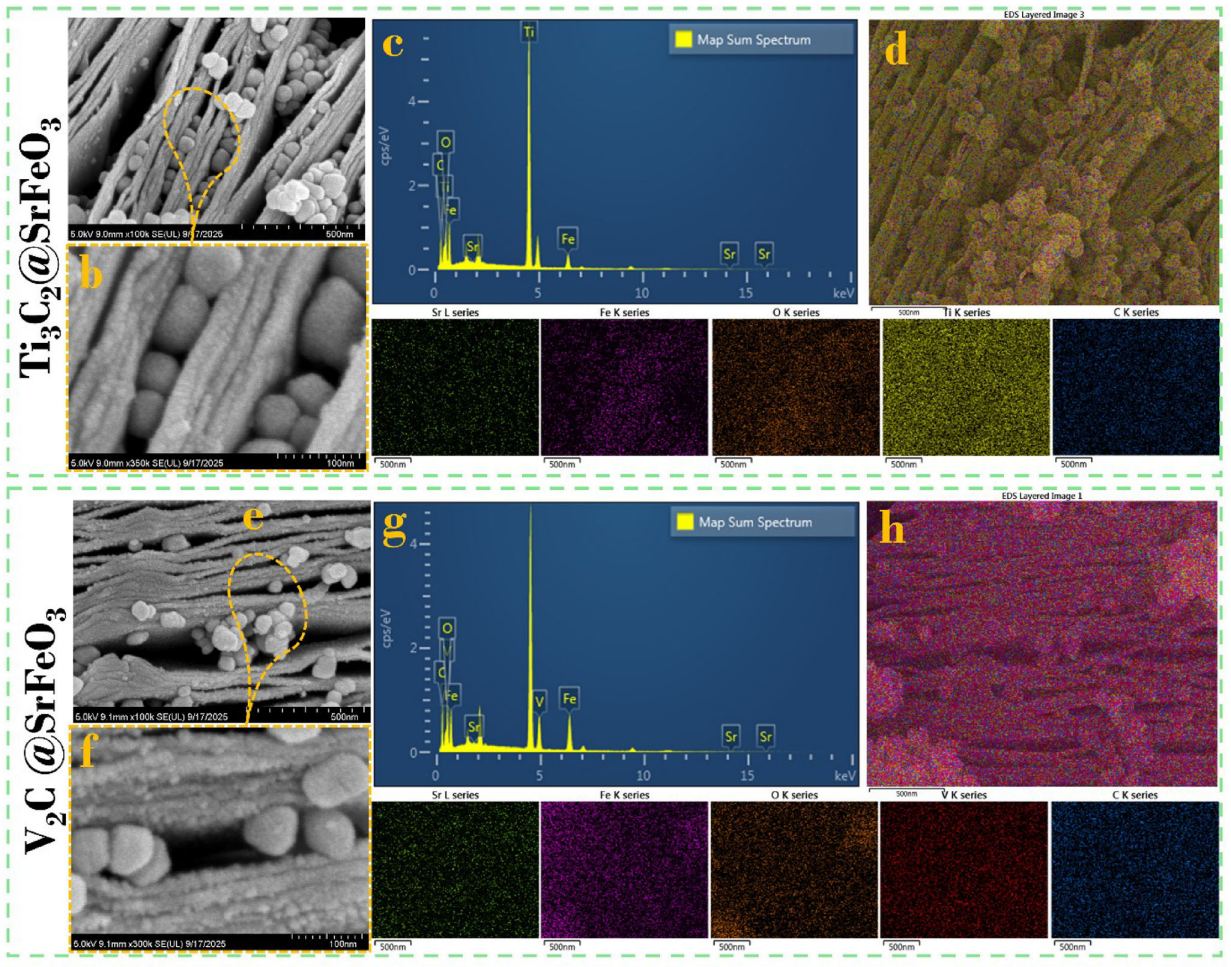
(a, b) SEM images of the Ti_3_C_2_T*_x_* @SrFeO_3_ hybrid. (c) EDS spectrum and (d) corresponding elemental maps of Sr, Fe, O, Ti, and C for Ti_3_C_2_T*_x_* @SrFeO_3_. (e, f) SEM images of the V_2_CT*_x_* @SrFeO_3_ hybrid. (g) EDS spectrum and (h) corresponding elemental maps of Sr, Fe, O, V, and C for V_2_CT*_x_* @SrFeO_3_.

Field‐emission scanning electron microscopy (FESEM) reveals a distinct morphological evolution from the pristine MXenes to their hybrid perovskite nanocomposites. The exfoliated Ti_3_C_2_T*_x_* and V_2_CT*_x_* precursors display the characteristic “exploded accordion” morphology of delaminated MXenes, consisting of loosely stacked, multi‐layered flakes with intrinsic wrinkles and pores. In contrast, the pure SrFeO_3_ consists of agglomerated nanoparticles with a quasi‐spherical or elliptical “lotus‐seed” morphology and a broad size distribution (Figure S1a–c), confirming their sub‐micron dimensions. Following the composite fabrication, a profound structural transformation is observed. In the Ti_3_C_2_T*_x_* @SrFeO_3_ and V_2_CT_x_@SrFeO_3_ composites (Figure 2a–b; Figure S1d–i), well‐dispersed SrFeO_3_ nanoparticles (50–100 nm in diameter and 500–100 nm) becomes uniformly embedded within the interlayer galleries of the MXene sheets. This integration effectively pillars the MXene layers, preventing their restacking and creating an expanded, hierarchical three‐dimensional architecture with multiscale porosity. The hierarchical architecture mitigates MXene restacking, maximizing the active surface area and promoting rapid ion diffusion. This design creates a robust conductive network that synergistically combines the MXene's electrical conductivity with the perovskite's pseudocapacitive contributions, making it highly advantageous for supercapacitors. The elemental composition and spatial distribution of the synthesized Ti_3_C_2_T*_x_* @SrFeO_3_ and V_2_CT*_x_* @SrFeO_3_ composites were analyzed using Energy‐Dispersive x‐ray Spectroscopy (EDS). Quantitative analysis yielded the following weight percentages: for Ti_3_C_2_T*_x_* @SrFeO_3_ (Figure 2c), Sr ≈ 33.7%, Fe ≈ 18.7%, O ≈ 7.08%, Ti ≈ 8.02%, and C ≈ 10.2%; for V_2_CT*_x_* @SrFeO_3_ (Figure 2g), Sr ≈ 31.7%, Fe ≈ 17.7%, O ≈ 9.08%, V ≈ 8.02%, and C ≈ 10.2%. Corresponding elemental mapping (Figure 2d,h) confirmed the homogeneous spatial distribution of all constituent elements Sr, Fe, O, Ti, and C for Ti_3_C_2_T*_x_* @SrFeO_3_, and Sr, Fe, O, V, and C for V_2_CT*_x_* @SrFeO_3_ across the analyzed regions. The pristine SrFeO_3_ (SFO) precursor was similarly characterized, with quantitative EDS (Figure S2a) revealing an atomic composition of Sr (8.6%), Fe (12.3%), and O (38.0%), alongside a significant carbon signal (41.1 at%) attributed to the conductive substrate. Elemental maps (Figure S2b–e) confirmed the uniform co‐localization of Sr, Fe, and O, verifying the successful formation of phase‐pure SFO nanoparticles. Collectively, these results confirm the successful integration and uniform dispersion of the SrFeO_3_ phase within the MXene matrix for both composite materials.

**FIGURE 3:**
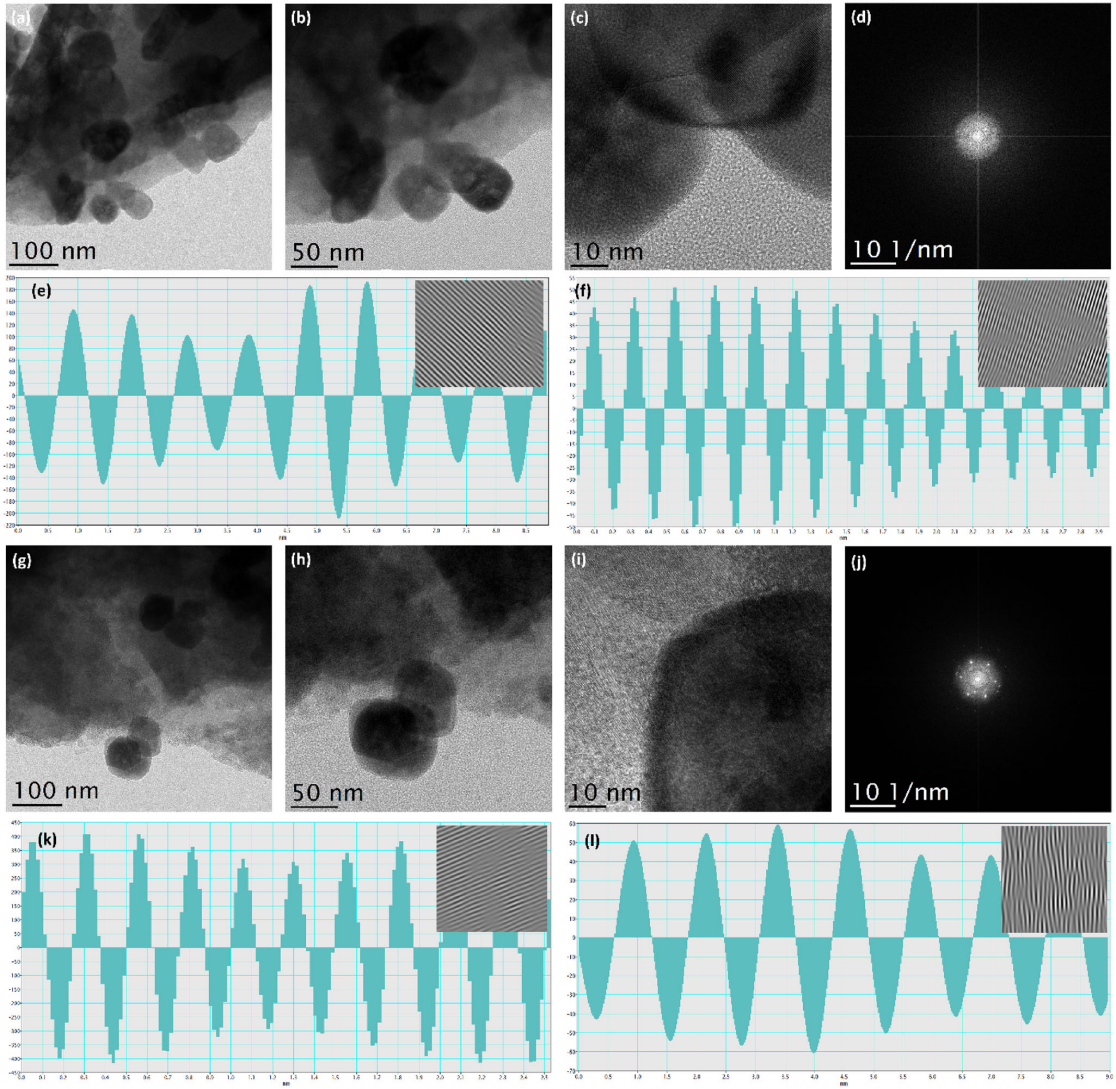
TEM images of the (a–f) Ti_3_C_2_T*_x_* @SrFeO_3_ and (g–l) V_2_CT*_x_* @SrFeO_3_ hybrids at different magnifications.

The microstructure of the Ti_3_C_2_T*_x_* @SrFeO_3_ and V_2_CT*_x_* @SrFeO_3_ composites were thoroughly investigated utilizing Transmission Electron Microscopy (TEM). Figure 3a–f presents the TEM images of the Ti_3_C_2_T*_x_* @SrFeO_3_ composites. The low‐magnification TEM image depicted in Figure 3a reveals the characteristic two‐dimensional sheet‐like morphology of the delaminated MXene hosts, specifically Ti_3_C_2_T*_x_*, which effectively serve as a structural scaffold for the composite. In the darker regions of the TEM image, exemplified in Figure 3b, one can observe the presence of SrFeO_3_ nanoparticles, while the lighter areas correspond to the MXene surfaces, indicating a clear contrast in composition. Upon examination of the high magnification images illustrated in Figure 3c, distinct features are observable, showcasing the agglomeration of SrFeO_3_ nanoparticles extensively decorating the surface of the Ti_3_C_2_T*_x_*. This decoration is indicative of strong interfacial interactions that enhance the composite's potential functional properties. The Fast Fourier Transform (FFT) profile of the Ti_3_C_2_T*_x_* @SrFeO_3_ composites, shown in Figure 3d, provides further insights into the crystallographic orientations present within the composite material. In Figure 3e, the exploration of lattice profile fringes (with an inset providing the corresponding inverse FFT) reveals a *d*‐spacing of approximately 0.97 nm associated with the Ti_3_C_2_T*_x_* MXene. This measurement is characteristic of the low‐angle plane within the layered structure of Ti_3_C_2_T*_x_*. Moreover, the lattice fringes observed with an interplanar spacing of 0.27 nm are attributed to the SrFeO_3_ nanoparticles, which further corroborates the composite's structural integrity. Similarly, Figure 3g‐l displays the TEM images for the V_2_CT*_x_* @SrFeO_3_ composites. The low‐magnification images, as seen in Figure 3g–h, elucidate the configuration of accumulated grains adorning the MXene surface. High magnification images, particularly shown in Figure 3i, again illustrate the prominent agglomeration of SrFeO_3_ nanoparticles on the V_2_CT*_x_* MXene surface, with their FFT analysis in Figure 3j outlining the polycrystalline nature of the material. In Figure 3k, the observed lattice fringes with a measured interplanar spacing of approximately 0.24 nm relate specifically to the (110) crystallographic plane of SrFeO_3_. Concurrently, distinct lattice fringes displaying a larger *d*‐spacing of around 1.26 nm are documented in Figure 3l, which emphasize the low‐angle plane characteristics inherent to the layered V_2_CT*_x_* MXene. These comprehensive analyses underscore the intricate structural relationships within the composites and their potential applications in energy storage uses.

**FIGURE 4:**
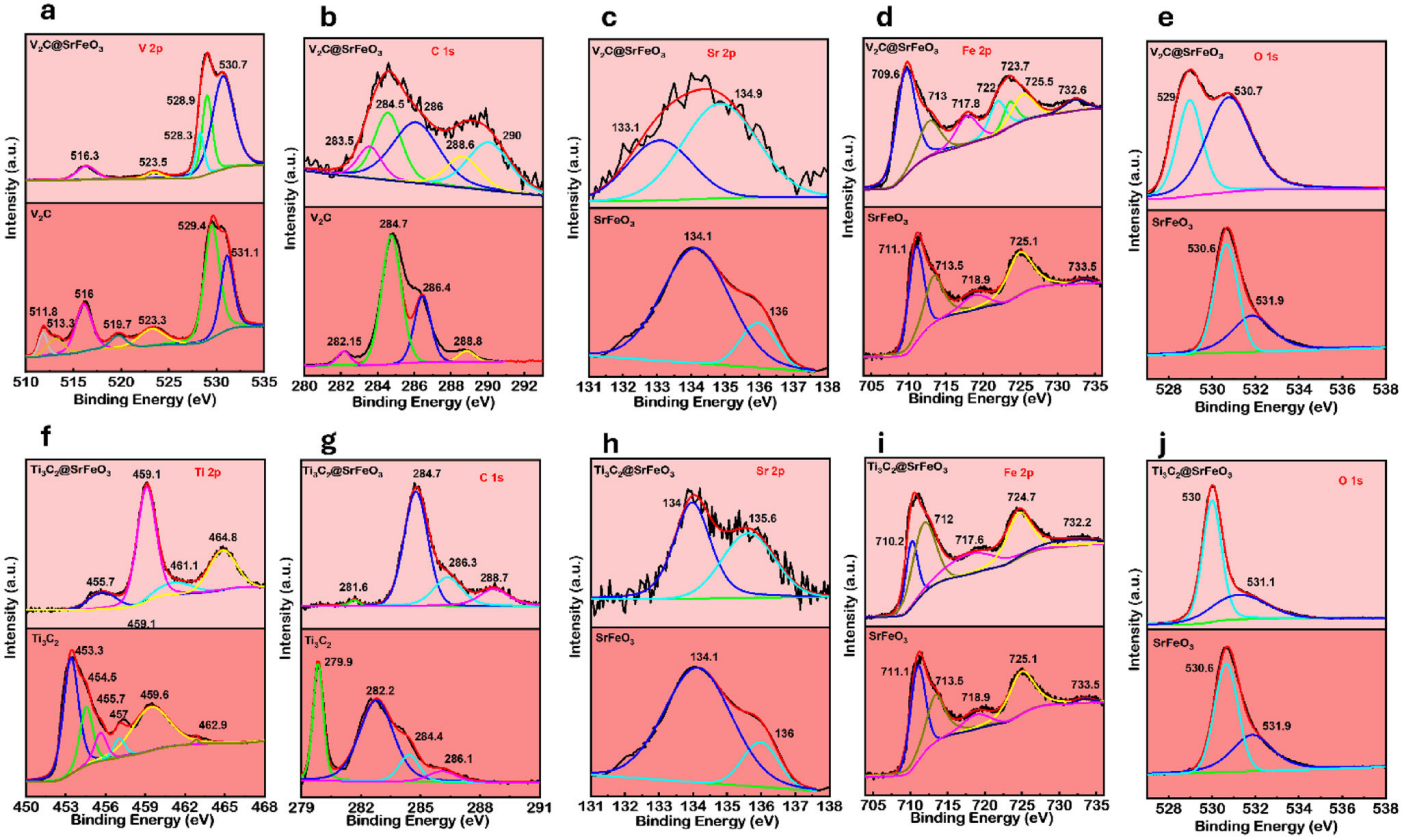
High‐resolution XPS core‐level spectra of the (a–e) V_2_CT*_x_* @SrFeO_3_ and (f–j) Ti_3_C_2_T*_x_* @SrFeO_3_ hybrids. Specific peaks for each: (a, f) V 2p and Ti 2p; (b, g) C 1s; (c, h) Sr 3d; (d, i) Fe 2p; (e, j) O 1s.

The surface chemical composition and oxidation states of the synthesized Ti_3_C_2_T*_x_* @SrFeO_3_ and V_2_CT*_x_* @SrFeO_3_ composites were analyzed by x‐ray photoelectron spectroscopy (XPS) (Figure 4). Survey scans (Figure S3) confirmed the presence of all constituent elements: Sr, Fe, O, Ti, and C for Ti_3_C_2_T*_x_* @SrFeO_3_, and Sr, Fe, O, V, and C for V_2_CT*_x_* @SrFeO_3_. For the V_2_CT*_x_* @SrFeO_3_ composite, the V 2p spectrumexhibits signatures characteristic of the V_2_CT*_x_* MXene, with peaks at 516.3 and 523.5 eV assigned to V–C bonds and components at 528.9 and 530.7 eV attributed to V–O surface functionalities. The Fe 2p spectrum was deconvoluted into contributions from Fe^2^^+^ (peaks at 713.8–717.8 and 723.7 –725.5 eV) and Fe^3^^+^ (peaks at 709.9–713 and 732.6 eV), where the Fe^3^^+^ 2p_3/2_ peak at 710.8 eV confirms the perovskite structure [28, 29]. The C 1s spectrum reveals oxygenated functional groups from the MXene at binding energies of 283.5 (C–C), 284.3, 286.9 (C–O/O–C–O), and 288.8–290 eV (O–C = O), which differ from the carbon environment in pure SrFeO_3_. The Sr 3d spectrum shows Sr^2^^+^ states at 133.1 (3d_5/2_) and 134.9 eV (3d_3/2_), slightly shifted from the positions in pristine SrFeO_3_ (134.1 and 136.0 eV). The O 1s spectrum is fitted with a major lattice oxygen component (O^2^^−^) at 529.3 eV and a surface hydroxyl/adsorbed water component at 530.7 eV [30]. Similarly, for the Ti_3_C_2_T*_x_* @SrFeO_3_ composite, the Ti 2p spectrum was deconvoluted into three doublets. Peaks at 455.7 (Ti 2p_3/2_) and 459.1 eV (Ti 2p_1/2_) correspond to Ti–C bonds, confirming the MXene core. A doublet at 461.1 (Ti 2p_3/2_) and 464.8 eV (Ti 2p_1/2_) is assigned to Ti–O bonds from surface terminations (T*_x_*) or oxidation, compared to 453.3–457 (Ti 2p_3/2_) and 459.6–462.9 eV (Ti 2p_1/2_) in pristine Ti_3_C_2_T*_x_*. The C 1s spectrum shows contributions at 281.6 (Ti–C), 284.7 (adventitious C–C), 286.3 (C–O/O–C–O), and 288.8 eV (O–C = O). For pristine Ti_3_C_2_T*_x_*, the C 1s peaks appear at 279.2 (Ti–C), 282.2 (C–C), 284.4, and 286.1 eV (C–O/O–C–O). The Sr 3d spectrum exhibits Sr^2^^+^ states at 134.0 (3d_5/2_) and 135.6 eV (3d_3/2_), slightly shifted from pristine SrFeO_3_ (134.1 and 136.0 eV). The Fe 2p spectrum presents a broadened profile deconvoluted into Fe^2^^+^ (711.1–712.8 eV) and Fe^3^^+^ (∼724 eV) contributions, with a satellite peak confirming the perovskite phase; for pure SrFeO_3_, Fe^2^^+^ appears at 711.1–713.5 eV and Fe^3^^+^ at 725.1 eV. The O 1s spectrum was fitted with lattice oxygen (O^2^^−^) at 530.0 eV and a surface component (hydroxyl/adsorbed water) at 531.1 eV, compared to 530.6 and 531.9 eV in pure SrFeO_3_. The observed binding‐energy shifts and the coexistence of Fe^2^^+^/Fe^3^^+^ states indicate strong interfacial coupling between the MXene and perovskite phases.

### 3.2 Supercapacitor Properties

#### 3.2.1 Half Cell Measurement

**FIGURE 5:**
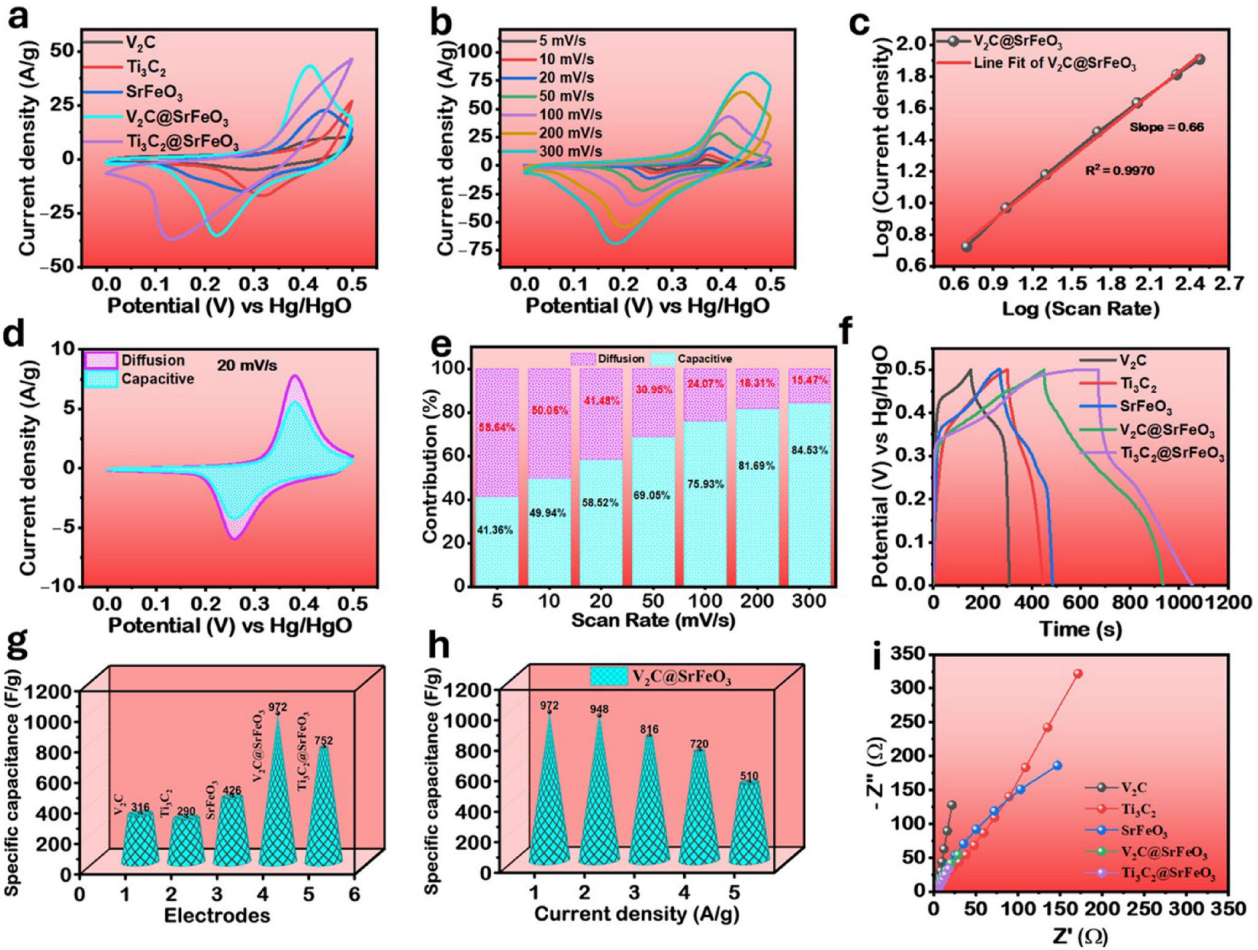
Electrochemical performance of pristine and hybrid electrodes in a full‐cell configuration. (a) CV profiles of V_2_C, Ti_3_C_2_, SrFeO_3_, V_2_CT*_x_* @SrFeO_3_, and Ti_3_C_2_T*_x_* @SrFeO_3_ at 100 mV s^−^^1^. (b) CV profiles of V_2_CT*_x_* @SrFeO_3_ at scan rates from 5 to 300 mV s^−^^1^. (c) Logarithmic plot of anodic peak current (i) versus function of scan rates (v) for V_2_CT*_x_* @SrFeO_3_. (d) Capacitive (shaded) and diffusion‐controlled contributions to the total CV area of V_2_CT*_x_* @SrFeO_3_ at a selected scan rate. (e) Percentage of the capacitive contribution for V_2_CT*_x_* @SrFeO_3_ at various scan rates. (f) GCD profiles at 1 A g^−^^1^, and (g) Specific capacitance of V_2_C, Ti_3_C_2_, SrFeO_3_, V_2_CT*_x_* @SrFeO_3_, and Ti_3_C_2_T*_x_* @SrFeO_3_. (h) Specific capacitance of V_2_CT*_x_* @SrFeO_3_ at various current densities. (i) Nyquist plot of V_2_C, Ti_3_C_2_, SrFeO_3_, V_2_CT*_x_* @SrFeO_3_, and Ti_3_C_2_T*_x_* @SrFeO_3_.

Electrochemical characterization of SrFeO_3_, Ti_3_C_2_T*_x_*, V_2_CT*_x_* MXenes and their hybrid composites (Ti_3_C_2_T*_x_* @SrFeO_3_ and V_2_CT*_x_* @ SrFeO_3_) was conducted in a three‐electrode system with a 1 M KOH electrolyte. Comparative cyclic voltammetry (CV) curves for SrFeO_3_, Ti_3_C_2_T*_x_*, V_2_CT*_x_*, Ti_3_C_2_T*_x_* @SrFeO_3,_ and V_2_CT*_x_* @SrFeO_3_ at a scan rate of 100 mV s^−^^1^ within a 0–0.5 V window all exhibited distinct redox peaks, indicating a charge‐storage mechanism dominated by pseudocapacitive faradaic reactions as shown in Figure 5a. Among the tested materials, the V_2_CT*_x_* @SrFeO_3_ hybrid demonstrated a substantially enhanced electrochemical response, evidenced by its largest CV integrated area and most pronounced redox peak intensity. This superior performance is attributed to a synergistic interplay between the components: the SrFeO_3_ perovskite provides characteristic Fe^2^^+^/Fe^3^^+^ redox activity (Fe^3^^+^–O + e^−^ + OH^−^ ⇌ Fe^2^^+^–O–OH), while the V_2_CT*_x_* MXene contributes high electrical conductivity and additional pseudocapacitance from vanadium cation transitions (e.g., V^3^^+^/V^4^^+^). The MXene's layered mesoporous structure further facilitates electrolyte ion transport, and the strong interfacial electronic coupling at the Fe–V interface optimizes charge‐transfer kinetics and redox reversibility. Rate capability and reactivity were further examined through CV measurements across scan rates from 5 to 300 mV s^−^^1^ (Figure 5b; Figures S4a, S5a, S6a, and S7a) for V_2_CT*_x_*, Ti_3_C_2_T*_x_*, SrFeO_3_, V_2_CT*_x_* @SrFeO_3,_ and Ti_3_C_2_T*_x_* @SrFeO_3_. The persistence of well‐defined redox features with only minimal potential shifts from 5 to 300 mV s^−^^1^ underscores the hybrid's excellent rate performance and highly efficient faradaic processes. This comprehensive analysis establishes the V_2_CT*_x_* @SreFO_3_ composite as a highly promising electrode material, significantly outperforming its individual constituents and the Ti_3_C_2_T*_x_* @SrFeO_3_ analogue.

The overall charge storage kinetics can be categorized into diffusion‐controlled (battery‐like) and surface‐controlled (pseudocapacitive) processes. The charge storage mechanism of the prepared electrodes was analyzed using the power‐law relationship between peak current (*i_p_*) and scan rate (ν):

(2) $$i_{p}=a\nu^{b}$$

where *a* and *b* are adjustable parameters. The exponent *b* is derived from the slope of log(*i_p_*) versus log(ν). A *b*‐value near 0.5 indicates diffusion‐controlled kinetics, while a value approaching 1 suggests surface‐controlled pseudocapacitive behavior.

For the SreFO_3_, V_2_CT*_x_* @SreFO_3_ and Ti_3_C_2_T*_x_* @SrFeO_3_ electrode, the *b*‐value of the anodic peak was calculated to be 0.64, 0.66 and 0.70 (Figure 5c; Figure S8a,b), indicating that the redox reactions are predominantly influenced by diffusion. Further quantitative analysis was performed by separating the current response at each potential into capacitive and diffusion‐controlled contributions using Dunn's method [31]:

(3) $$i\left(\nu \right)=k_{1}\;\nu +k_{2}\nu^{1/2}$$

where *k*_1_ν represents the capacitive current and *k*_2_ν^1/2^ corresponds to diffusion‐limited pseudocapacitance. By plotting *i*/ν^1/2^ versus ν^1/2^, the constants *k*_1_ and *k*_2_ can be obtained from the slope and intercept, respectively [32, 33]. At different scan rates of 5 ∼ 300 mV s^−^^1^, the diffusion‐controlled contributions for V_2_CT_x_@SFO were 58.64%, 50.05%, 41.48%, 30.95%, 24.07%, 18.31%, and 15.47% respectively, while capacitive contributions accounted for the remaining 41.36%, 49.94%, 58.52%, 69.05%, 75.93%, 81.69%, and 84.53%, (Figure ‐5d,e). The high diffusion contribution confirms fast redox kinetics and a significant pseudocapacitive behavior, underscoring the efficient charge storage facilitated by the hybrid electrode structure.

The GCD profiles of SrFeO_3_, Ti_3_C_2_T*_x_*, V_2_CT*_x_* MXenes, and their hybrid composites (Ti_3_C_2_T*_x_* @SrFeO_3_ and V_2_CT*_x_* @SreFO_3_) were evaluated between 0 and 0.5 V as shown in Figures S4b, S5b, S6b, S7b and 5f. The nonlinear curves for SreFO_3_‐based electrodes, especially V_2_CT*_x_* @SrFeO_3_, show defined plateaus, confirming a pseudocapacitive faradaic storage mechanism consistent with CV data. The V_2_CT*_x_* @SreFO_3_ hybrid exhibited a remarkable enhancement in specific capacitance. As shown in Figure 5g, the specific capacitance (C_s_) at 1 A g^−^^1^ was calculated to be 316, 290 and 426 F g^−^^1^ for pristine V_2_CT*_x_*, Ti_3_C_2_T*_x_*, SrFeO_3,_ respectively, while significantly higher values of 752 and 972 F g^−^^1^ were achieved for Ti_3_C_2_T*_x_* @SrFeO_3_ and V_2_CT*_x_* @SrFeO_3_. The remarkable enhancement observed for the hybrid electrodes is attributed to the synergistic integration of the highly conductive MXene framework with the redox‐active SrFeO_3_ perovskite, which facilitates rapid charge transport and provides abundant electrochemically accessible active sites.

Upon increasing the current density from 1 to 5 A g^−^^1^, V_2_CT*_x_* @SrFeO_3_ retained a high capacitance of 510 F g^−^^1^ from an initial 972 F g^−^^1^ (Figure 5h), whereas Ti_3_C_2_T*_x_* @SrFeO_3_ delivered 380 from 752 F g^−^^1^ (Figure S7c). In contrast, V_2_CT*_x_*, Ti_3_C_2_T*_x_*, and SrFeO_3_ exhibited comparatively lower capacitance retention, from 316 to 190 F g^−^^1^ (Figure S4c), 290 to 170 F g^−^^1^ (Figure S5c), and 426 to 180 F g^−^^1^ (Figure S6c), respectively. The enhanced rate capability of the hybrid composites highlights the beneficial role of MXene in accelerating electron transport and improving electrolyte‐ion diffusion.

The Ragone plot analysis further demonstrates the energy‐storage superiority of the hybrid systems (Figures S4d, S5d, S6d, S7d, and S9). V_2_CT*_x_* @SrFeO_3_ delivered an energy density of 33.75 Wh kg^−^^1^ at a power density of 0.25 kW kg^−^^1^ and retained 17.7 Wh kg^−^^1^ at 1.25 kW kg^−^^1^. Similarly, Ti_3_C_2_T*_x_* @SrFeO_3_ achieved energy densities of 26.11 and 13.19 Wh kg^−^^1^ at power densities of 0.25 and 1.25 kW kg^−^^1^, respectively. Compared with pristine V_2_CT*_x_*, Ti_3_C_2_T*_x_*, and SrFeO_3_, which exhibited energy densities of 10.97, 10.06, and 14.79 Wh kg^−^^1^ at 0.25 kW kg^−^^1^ power densities. Ti_3_C_2_T*_x_* @SrFeO_3_ and V_2_CT*_x_* @SrFeO_3_ demonstrate substantial performance enhancements. These findings clearly indicate that the incorporation of MXene not only improves specific capacitance and rate capability but also significantly boosts the energy‐storage characteristics of the SrFeO_3_‐based electrode system.

The superior electrochemical performance of V_2_CT*_x_* @SrFeO_3_ originates from multiple synergistic mechanisms: (1) The V_2_C and Ti_3_C_2_T*_x_* MXene framework provides exceptional electrical conductivity (≈10^3^ S cm^−^^1^) compared to SrFeO_3_, enabling faster electron transport throughout the electrode matrix [34, 35]; (2) The unique core‐shell architecture creates a 3D conductive network that maximizes active site utilization while maintaining structural integrity during redox processes; (3) V_2_C's intrinsically larger interlayer spacing (≈1.2 nm vs. ≈ 0.95‐1.0 nm for Ti_3_C_2_T*_x_* under comparable conditions) facilitates rapid OH^−^ ion diffusion, as evidenced by the reduced voltage drops in GCD curves [36, 37]; (4) Strong electronic coupling at the V_2_CT*_x_* @SrFeO_3_ interface promotes charge transfer kinetics. This is augmented by vanadium's multiple oxidation states (e.g., V^3^^+^/V^4^^+^), which contribute additional pseudocapacitance beyond the primary Fe^2^^+^/Fe^3^^+^ redox couple of SrFeO_3_; (5) The superior performance originates from a synergistic interplay between the V_2_CT*_x_* and SrFeO_3_ components, which enhances cumulative redox kinetics evidenced by extended discharge plateaus and from a robust 3D conductive architecture that maximizes active site accessibility and cycling stability.

Cyclic stability a key metric for practical electrode materials was assessed through long‐term galvanostatic charge‐discharge (GCD) testing. After 5000 cycles at 3 A g^−^^1^, capacitance retention reached 91.2% for V_2_CT*_x_* @SrFeO_3_ and 93.9% for Ti_3_C_2_T*_x_* @SrFeO_3_, compared to only 69.5% for pristine SrFeO_3_ (Figure S10). This enhancement is linked to the distinct composite architecture of the MXene hybrids. At elevated current densities, Ti_3_C_2_T*_x_* @SrFeO_3_ exhibits particularly strong rate capability, retaining 93.9% of its initial capacitance even at 3 A g^−^^1^ after 5000 cycles. Its exceptional long‐term cycling stability and high‐rate performance in a three‐electrode setup establish Ti_3_C_2_T*_x_* @SrFeO_3_ as a highly promising candidate for advanced energy storage, where high capacitance and rapid charge‐discharge are critical.

The electrochemical impedance spectra (Figure 5i) reveal that V_2_CT*_x_* @SrFeO_3_ exhibits the lowest charge‐transfer resistance (Rct = 1.9 Ω), significantly outperforming Ti_3_C_2_T*_x_* @SrFeO_3_ (2.2 Ω) and pristine SrFeO_3_ (121 Ω). This substantial improvement in charge‐transfer kinetics is attributed to several synergistic properties of the V_2_CT*_x_* MXene: (1) its metallic conductivity (σ ≈ 10^3^ S/m) establishes efficient electron transport pathways; (2) oxygen/fluorine surface terminations create defect‐mediated charge‐transfer sites; (3) expanded interlayer spacing (∼12 Å) enhances electrolyte ion accessibility; [38] and (4) redox‐active vanadium centers enable low‐energy pseudocapacitive charge storage [39]. These synergistic effects collectively explain V_2_CT*_x_* @SrFeO_3_ superior conductivity, rapid charge‐transfer kinetics, and enhanced high‐rate performance.

The integration of Ti_3_C_2_T*_x_* and V_2_CT*_x_* MXenes with 0D SrFeO_3_ nanoparticles via a straightforward mechanical mixing route presents a scientifically compelling strategy for advancing supercapacitor performance. This approach effectively creates a synergistic composite that leverages the distinct advantages of each material while mitigating their individual limitations. The MXene components, specifically Ti_3_C_2_T*_x_* and V_2_CT*_x_*, serve as a highly conductive and capacitive scaffold, providing a pathway for rapid electron transport and contributing substantial pseudocapacitance through their surface redox‐active terminations. However, a primary challenge for MXenes is their tendency to restack, which reduces active surface area and impedes ion access. This is where the 0D SrFeO_3_ nanoparticles play a critical dual role: they function as robust pseudocapacitive boosters, leveraging the reversible Fe^3^^+^/Fe^4^^+^ redox couple of the perovskite structure to significantly enhance Faradaic charge storage and energy density, while simultaneously acting as physical spacers between the MXene sheets. This spacer effect prevents restacking, ensuring electrolyte penetration and maximizing the utilization of the entire electrode architecture. The mechanical mixing method is key to this success, as it is a simple, scalable technique that preserves the intrinsic properties of both materials, creating a “bridged” network where the MXene matrix wires up the otherwise less‐conductive SrFeO_3_ nanoparticles, and the nanoparticles create enhanced ion transport pathways. Ultimately, this composite elegantly combines the fast kinetics and conductivity of MXenes with the high theoretical capacitance of SrFeO_3_, resulting in a hybrid supercapacitor electrode engineered for high energy density, exceptional power density, and prolonged cycle life.

The post‐cycling FESEM images of the V_2_CT*_x_* @SrFeO_3_ and Ti_3_C_2_T*_x_* @SrFeO_3_ hybrid electrodes acquired at different magnifications (Figure S11) reveal that the overall microstructure remains well preserved after extended charge–discharge cycling. Both electrodes retain their interconnected porous framework, while the active particles remain firmly anchored on the conductive carbide matrix without evidence of severe aggregation or structural disintegration. Notably, the characteristic layered morphology of the MXene component and the uniformly dispersed SrFeO_3_ particles are still clearly distinguishable, indicating excellent morphological stability during repeated electrochemical operation.

Furthermore, no significant surface cracking, particle pulverization, or delamination is observed, demonstrating the strong interfacial integration between the carbide and perovskite phases. Preserved architecture facilitates continuous electrolyte penetration and efficient electron/ion transport, thereby minimizing structural degradation during long‐term cycling. The excellent retention of the electrode morphology corroborates the outstanding cycling stability and capacitance retention observed electrochemically, confirming the robustness of the hybrid electrode design for durable supercapacitor applications.

#### 3.2.2 Full Cell Measurement

**FIGURE 6:**
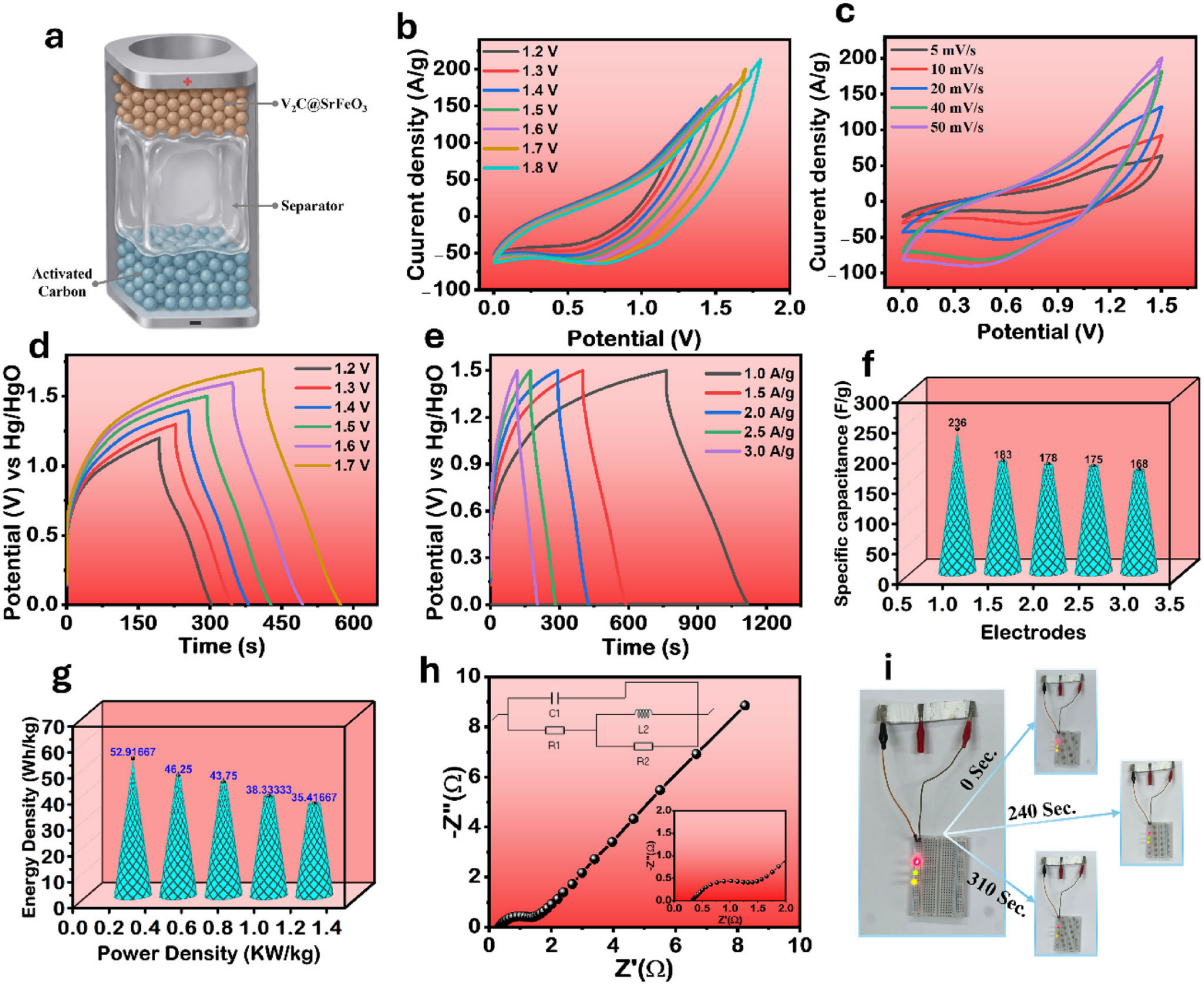
Electrochemical performance of a V_2_CT*_x_* @SrFeO_3_‐based hybrid ASC device. (a) Schematic of the asymmetric cell configuration. (b) CV profiles at a scan rate of 20 mV s^−^^1^ with varying potential windows (1.2–1.8 V). (c) CV profiles at different scan rates within the optimal window. (d) GCD profiles at 20 mV s^−^^1^ with varying potential windows (1.2–1.7 V). (e) GCD profiles at different current densities within the optimal window. (f) Specific capacitance of the HSC device as a function of current density. (g) Ragone plot (energy density vs. power density). (h) EIS Nyquist plot. (i) Demonstration of two series‐connected devices powering an LED indicator.

To evaluate the practical applicability and functional utility of the V_2_CT*_x_* @SrFeO_3_ hybrid composite as a viable SC for modern electronics, we fabricated an all‐solid‐state ASC device. This assembly consisted of V_2_CT*_x_* @SrFeO_3_ as the positive electrode and activated carbon (AC) as the negative electrode, separated by a KOH/PVA gel electrolyte layer. The fabrication methodology and cross‐sectional architecture of the constructed ASC are schematically depicted in Figure 6a, respectively (see Supporting Information for detailed procedures). Figure 6b shows CV profiles of the ASC cell, measured at a scan rate of 10 mV s^−^^1^, across different potential windows (1.2 to 1.8 V). The current response and enclosed area of the CV curves increased as the operational voltage was extended to 1.7 V. However, beyond this potential, an undesirable peak associated with oxygen evolution appears in the voltammogram, indicating parasitic side reactions. Consequently, the optimal working window was fixed at 0 to +1.7 V for all subsequent electrochemical characterization. To assess the device's rate performance, CV measurements were performed at scan rates from 10 to 50 mV s^−^^1^ (Figure 6c). The CV profiles maintain their characteristic shape even at the highest rate of 50 mV s^−^^1^, confirming the ASC's excellent rate capability. This stability is further corroborated by GCD curves (Figure 6d). The GCD profile at 1.8 V displays a tailing feature at the end of the charging segment, a signature of side reactions, which reinforces the selection of the 1.7 V operational limit. The GCD profiles of the V_2_CT*_x_* @SrFeO_3_/NF//AC device, measured between 0 and 1.7 V at current densities from 1 to 3 A g^−^^1^, are presented in Figure 6e. The nonlinear shape of the curves confirms the hybrid charge‐storage mechanism, involving both electric double‐layer (EDLC) and pseudocapacitive contributions, as suggested by the CV analysis. This device demonstrates excellent rate capability, delivering specific capacitances of 236, 183, 178, 175, and 168 F g^−^^1^ at 0.5, 1.5, 2.0, 2.5, and 3.0 A g^−^^1^, respectively (Figure 6f). The corresponding Ragone plot (Figure 6g) highlights superior energy–power characteristics, with a high energy density of 52.91 Wh kg^−^^1^ at a power density of 0.25 kW kg^−^^1^, which remains as high as 35.41 Wh kg^−^^1^ at 1.25 kW kg^−^^1^. These values exceed those of many previously reported perovskite‐ and MXene‐based supercapacitors, such as MXene–MnO_2_ (19.4 Wh kg^−1^ @ 1.5 kW kg^−1^) [40], Co@MXene‐ composite (26.6 Wh Kg^−1^@700 W Kg^−1^) [41], MXene‐PANI (38 Wh kg^−1^@ 808 W kg^−1^) [42], NiCo‐MOF/Ti_3_C_2_T*_x_* (39.5 Wh kg^−1^@562.5 W kg‐1) [43], MoS_2_@MXene // MXene (1.21 Wh kg^−1^ @ 54.45 W kg^−1^) [44], MnO_2_‐Co_3_O_4_@MXene‐CNF// Co‐PC@MXene‐CNF‐ FW‐QSS‐HSC (72.5 Wh kg^−1^ at 832.4 W kg^−1^) [45], NiCo_2_‐LDHs@MXene/rGO (65.3 Wh kg^−1^@ 700 W kg^−1^) [46] PANI/WO_3_/MXene (65.7 Wh kg^−1^@ 797.9 W kg^−1^) [47], Ti_3_C_2_T*_x_*/ACF (44.36 Wh kg^−1^@985 Wkg^−1^) [48], NiCo_2_O_4_/NiO/MXene (CNOT) (23.77 Wh Kg^−1^@1808.87 W Kg^−1^) [49], Ti_3_C_2_T*_x_*/Mo‐CuO (93.34 Wh Kg^−1^@3679.25 W Kg^−1^) [50], MnNbS‐MXene (35.77 Wh Kg^−1^@ 2800 W Kg^−1^) [51], NiCo_2_O_4_/ MXene/rGO (40.5 Wh kg^−1^ @ 1125.1 W kg^−1^) [52] as well as shown in the comparative analysis of Figure S12. Furthermore, the device exhibits remarkable long‐term stability, retaining 88.49% of its initial capacitance after 5000 cycles at 1 A g^−^^1^ (Figure S13). EIS reveals efficient charge transport, with a low equivalent series resistance (R*_s_*) of 1.71 Ω and a charge‐transfer resistance (Rc*_t_*) of 1.96 Ω (Figure 6h). The combined metrics of high energy and power density, exceptional rate capability, robust cycling stability, and low interfacial resistance establish the SFO@V_2_C/NF//AC asymmetric supercapacitor as a highly promising candidate for advanced energy storage applications. In Figure 6i, two ASCs connected in series power three parallel light‐emitting diodes (LEDs)‐blue, yellow, and red—each operating at 5 V. These LEDs remain illuminated for up to 290 , 300 , and 320 s, respectively. The superior performance demonstrated by the SFO@V_2_C/NF//AC ASC highlights its promising potential for asymmetric supercapacitor applications. The Ti_3_C_2_T*_x_* @SrFeO_3_ and V_2_CT*_x_* @SrFeO_3_ composites were prepared via a simple mechanical mixing method, integrating SrFeO_3_ nanoparticles with Ti_3_C_2_T*_x_* or V_2_CT*_x_* MXenes. In these composites, MXenes provide a highly conductive framework and intrinsic pseudocapacitance, while SrFeO_3_ contributes additional Fe^3^^+^/Fe^4^^+^ redox activity. SrFeO_3_ also acts as an interlayer spacer, mitigating MXene restacking. This structural role improves electrolyte accessibility and increases the exposure of active sites. The resulting interconnected architecture enhances electrical conductivity, ion transport, and structural stability, ultimately delivering high energy density, robust rate capability, and excellent cycling performance in supercapacitors.

## 4 Conclusion

In summary, we have successfully synthesized and characterized a high performance V_2_CT*_x_* @SrFeO_3_ and Ti_3_C_2_T*_x_* @SrFeO_3_ hybrid electrode. Electrochemical evaluation confirms that this composite significantly outperforms its individual constituents and the Ti_3_C_2_T*_x_* @SrFeO_3_ analogue, demonstrating a superior specific capacitance of 972 F g^−^^1^ at 1 A g^−^^1^ and enhanced rate capability. This performance stems from synergistic effects: the conductive V_2_CT*_x_* MXene acts as a robust, high‐surface‐area scaffold for rapid electron transport and ion diffusion, while the SrFeO_3_ perovskite supplies high‐capacity redox activity through the Fe^2^^+^/Fe^3^^+^ couple. Charge storage kinetics, quantified via power‐law and deconvolution analyses, reveal a hybrid mechanism with a favorable capacitive contribution, enabling high‐rate performance. Further redox activity of vanadium cations and strong interfacial coupling optimize the overall charge storage. The promise of this architecture is validated in a full ASC device. The V_2_CT*_x_* @SrFeO_3_/NF//AC cell delivers a high energy density of 52.91 Wh kg^−^^1^ at 0.25 kW kg^−^^1^ and exhibits outstanding cycling stability, retaining 88.49% of its initial capacitance after 5000 cycles at 10 A g^−^^1^. Therefore, the V_2_CT*_x_* @SrFeO_3_ hybrid presents a highly effective strategy for developing next‐generation energy storage devices that simultaneously achieve high energy and power densities.

## Author Contributions

**Sayed Zafar Abbas**: software, investigation, validation. **Hyun‐Seok Kim**: validation, visualization. **Zulfqar Ali Sheikh**: writing – original draft, methodology, data curation, conceptualization, formal analysis. **Faisal Shahzad**: methodology, formal analysis, visualization. **Sajjad Hussain**: writing – review and editing, visualization, supervision, formal analysis, investigation. **Jongwan Jung**: formal analysis, supervision, visualization, funding acquisition. **Doyoung Byun**: formal analysis, visualization. **Shahzaib Ali**: methodology, validation. **Dhanasekaran Vikraman**: formal analysis, data curation.

## Funding

This research was supported by the Basic Science Research Program through the National Research Foundation of Korea (NRF) funded by the Ministry of Education (2020R1A6A1A03043435). Additional support was provided by the Korea Basic Science Institute (National research facilities and equipment center) grant funded by the Ministry of Education (2022R1A6C101A774). This work was also supported by the Industrial Strategic Technology Development Program (20026691), which is funded by the Ministry of Trade, Industry & Energy (MOTIE), Korea.

## Conflicts of Interest

The authors declare no conflicts of interest.

## Supporting information




**Supporting File**: Smll74641‐sup‐0001‐SuppMat.docx.

## Data Availability

Data sharing not applicable to this article as no datasets were generated or analyzed during the current study.
