# Supplementary material for: Synergistic Integration of SrFeO3 Nanoparticles With Exfoliated MXene (Ti3C2T x /V2CT x ) Nanosheets for High Performance Supercapacitors
Source: Small. 2026 Aug 4;22(51):e74641. doi: 10.1002/smll.74641 (PMC13568846; doi:10.1002/smll.74641)
Supplement: Supplementary file 1 [file SMLL-22-e74641-s001.docx]

**Supporting Information**

**Synergistic Integration of SrFeO₃ Nanoparticles with Exfoliated MXene (Ti_3_C_2_Tₓ/V₂CTₓ) Nanosheets for High-Performance Supercapacitors**

Zulfqar Ali Sheikh^a,b^, Shahzaib Ali^c^, Sayed Zafar Abbas^a,b^, Dhanasekaran Vikraman^d^, Faisal Shahzad^e^, Doyoung Byun^c^, Hyun-Seok Kim^d^, Sajjad Hussain^a,b^*, Jongwan Jung^a,b^**

^a^ Hybrid Materials Center (HMC), Sejong University, Seoul 05006, South Korea

^b^ Department of Nanotechnology and Advanced Materials Engineering, Sejong University, Seoul 05006, South Korea

^c^ Department of Mechanical Engineering, Sungkyunkwan University, Suwon, Gyeonggi-do, South Korea

^d^ Division of Electronics and Electrical Engineering, Dongguk University-Seoul, Seoul 04620, South Korea

^e^ Research and Innovation Center for Graphene and 2D Materials (RIC2D), Khalifa University, P.O. Box 127788, Abu Dhabi, United Arab Emirates

*Corresponding author’s Email: [hussain@sejong.ac.kr](mailto:v.j.dhanasekaran@gmail.com) , [jwjung@sejong.ac.kr](mailto:jwjung@sejong.ac.kr)

**Figure S1.** FESEM micrography with different resolutions of (a–c) SrFeO_3_; (d–f) Ti_3_C_2_@SrFeO_3_ and (g–i) V_2_C@SrFeO_3_ hybrid composites.

**Figure S2.** Elemental composition and distribution of the SrFeO_3_ composite. (a) EDS spectrum with corresponding elemental atomic percentages. (b) Elemental mapping showing the spatial distribution of Sr, Fe, O, and C.

 **Figure S3.** XPS survey spectra of pristine and hybrid composites. (a) Pristine V₂C MXene and the V_2_C@SrFeO_3_ hybrid composite. (b) Pristine Ti₃C₂ MXene and the Ti_3_C_2_@SrFeO_3_ hybrid composite.

**Figure S4:** Electrochemical performance of the V_2_C electrode. (a) CV curves at scan rates from 5 to 300 mV s⁻¹. (b) GCD profiles at current densities from 1 to 5 A g⁻¹. (c) Specific capacitance as a function of current density. (d) Ragone plot relating energy density versus power density for the V_2_C.

**Figure S5:** Electrochemical performance of the Ti_3_C_2_ electrode. (a) CV curves at scan rates from 5 to 300 mV s⁻¹. (b) GCD profiles at current densities from 1 to 5 A g⁻¹. (c) Specific capacitance as a function of current density. (d) Ragone plot relating energy density versus power density for the Ti_3_C_2_.

**Figure S6.** Electrochemical performance of the SrFeO_3_ electrode. (a) CV curves at scan rates from 5 to 300 mV s⁻¹. (b) GCD profiles at current densities from 1 to 5 A g⁻¹. (c) Specific capacitance as a function of current density. (d) Ragone plot relating energy density to power density.

**Figure S7.** Electrochemical performance of the Ti_3_C_2_@SrFeO_3_ electrode. (a) CV curves at scan rates from 5 to 300 mV s⁻¹. (b) GCD profiles at current densities from 1 to 5 A g⁻¹. (c) Specific capacitance as a function of current density. (d) Ragone plot relating energy density to power density.

**Figure S8.** (a-b) Total charge (Qₜ) versus the reciprocal square root of scan rate (v⁻¹/²) for SrFeO_3_ and Ti_3_C_2_@SrFeO_3_.

**Figure S9.** Ragone plot depicting the energy density versus power density for the V_2_C@SrFeO_3_ hybrid composite in a three-electrode half-cell configuration.

**Figure S10**. Stability performance of the three-electrode supercapacitor device based on SrFeO_3_, Ti_3_C_2_@SrFeO_3_ and V_2_C@SrFeO_3_.

**Figure S11:** Post-cycling FESEM micrographs of the Ti_3_C_2_@SrFeO_3_ and V_2_C@SrFeO_3_ hybrid electrodes recorded at different magnifications after prolonged electrochemical cycling.

**Table S1.** Half-cell supercapacitor performances of TMD and perovskite-based electrodes

| **Electrode materials** | **Electrolyte** | **Specific capacitance** | **Energy density** | **Power density** | **Capacitance retention (%)/cycles** | **Ref.** |
| --- | --- | --- | --- | --- | --- | --- |
| ***V_2_C@SrFeO_3_*** | ***1 M KOH*** | ***972 F·g^−1^ @ 1 A·g^−1^*** | ***33.75 Wh kg⁻¹*** | ***0.25 kW kg⁻¹*** | ***91.2/5000*** | ***In this study*** |
| ***Ti_3_C_2_@SrFeO_3_*** | ***1 M KOH*** | ***752 F·g^−1^ @ 1A·g^−1^*** | ***26.11 Wh kg⁻¹*** | ***0.25 kW kg⁻¹*** | ***93.9/5000*** | ***In this study*** |
| MoS_2_/MXene  nanohybrid | 3 M KOH | 583 F·g^-1^ @  1 A·g^-1^ | - | - | 82.5/3000 | [1] |
| MXene-NiCo_2_S_4_@NF | 3 M KOH | 1147.47 F·g^-1^ @ 1 A·g^-1^ | - | - | 80.4/3000 | [2] |
| WSe_2_@2D Ti_3_C_2_T*_x_* | 3 M KOH | 188.16 F·g^-1^ @ 1 A·g^-1^ | - | - | 80/10000 | [3] |
| MXene-rGO | 6 M KOH | 376 F·g^-1^ @ 1 A·g^-1^ | - | - | 97.3/8000 | [4] |
| Ti_2_NbC_2_T_x_  -β-borophene | 6 M KOH | 510 F·g^-1^ @ 1 A·g^-1^ | - | - | 92.7/5000 | [5] |
| VSe_2_/e-MXene/CNT | 6 M KOH | 182 F·g^-1^ @ 1 A·g^-1^ | - | - | 91.5/5000 | [6] |
| MXene/(PANI)/ZnO | 1 M H_2_SO_4_ | 651.96 F·g^-1^ @ 0.1 A·g^-1^ | - | - | 94.75/10000 | [7] |
| Ti_3_C_2_T_x_/MoS_2_ | 1 M H_2_SO_4_ | 595 F·g^-1^ @ 1 A·g^-1^ | - | - | 82/2000 | [8] |
| Ti_3_C_2_ MXene/  MoS | 1M H_2_SO_4_ | 679 F·g^-1^ @ 1 A·g^-1^ | - | - | 83.81/10000 | [9] |
| Nb_2_C/MoSe_2_ | 1 M (NH_4_)_2_SO_4_ | 252 F·g^-1^ @ 1 A·g^-1^ | - | - | 97/10000 | [10] |
| Modified MXene/AC | 1 M H_2_SO_4_ | 407 F·g^-1^ @ 5 mV s^−1^ | - | - | 93.13/5000 | [11] |
| MXene–MnO_2_ | 6 M KOH | 212 F·g^-1^ @ 1 A·g^-1^ | - | - | - | [12] |

To provide a comprehensive comparison with previously reported systems, a radar plot was constructed using normalized electrochemical performance metrics, including specific capacitance, energy density, power density, cycling stability, and capacitance retention (Figure S13). The comparison includes NiCoS/d-Ti₃C₂[13], NiMoO₄/Ti₃C₂Tₓ[14], V₂NTₓ [15], MXene@Ni–Mn LDH[16] Ni₁.₅Co₁.₅S₄-5@Ti₃C₂[17], and MXene/CuS [18] asymmetric supercapacitors. Among these reported devices, the V₂C@SrFeO₃/AC system exhibits a well-balanced electrochemical performance, delivering high specific capacitance and energy density while maintaining competitive cycling stability and capacitance retention, demonstrating its potential as a promising electrode material for advanced energy storage applications.

**Figure S12:** Comparison of the electrochemical performance of the V₂C@SrFeO₃//AC asymmetric supercapacitor device with recently reported MXene-based supercapacitor devices in terms of specific capacitance, energy density, power density, and cycling stability.

**Figure S13**. Long-term cycling stability of the V₂C-hybrid composite//activated carbon asymmetric supercapacitor.

**Reference:**

[1] B. Kirubasankar, M. Narayanasamy, J. Yang, M. Han, W. Zhu, Y. Su, S. Angaiah, C. Yan, Construction of heterogeneous 2D layered MoS2/MXene nanohybrid anode material via interstratification process and its synergetic effect for asymmetric supercapacitors, Applied Surface Science, 534 (2020) 147644.

[2] H. Li, X. Chen, E. Zalnezhad, K. Hui, K. Hui, M.J. Ko, 3D hierarchical transition-metal sulfides deposited on MXene as binder-free electrode for high-performance supercapacitors, Journal of Industrial and Engineering Chemistry, 82 (2020) 309-316.

[3] A. Manimekalai, S. Mohandoss, R. Venkatesan, A.A. Vetcher, K.P. Arunachalam, K.S. Velu, S. Sun, S.-C. Kim, Nanoflower-like 2D WSe_2_-wrapped 2D Ti_3_C_2_T_x_ MXene heterostructure for supercapacitor applications, Scientific Reports, 16 (2026) 14590.

[4] S. Mariam Varghese, R.B. Rakhi, Investigating the Electrochemical Properties of Ti3C2T x MXene-Carbon-Based Composites as Efficient Anode Materials for High-Performance Supercapacitors, ACS Applied Electronic Materials, 8 (2026) 3439-3452.

[5] Y.O. Yesilbag, F.N.T. Yesilbag, A. Huseyin, A.J.S. Salih, M. Ertugrul, 2D/2D Borophene/Ti_2_NbC_2_T_x_ MXene hybrids as advanced electrodes for supercapacitors, Chemical Engineering Journal, (2026) 176896.

[6] P. Siddu, S.R. KA, S. Radhakrishnan, S. Mun Jeong, C. Sekhar Rout, 3D ternary hybrid of VSe_2_/e‐MXene/CNT with a promising energy storage performance for high performance asymmetric supercapacitor, Batteries & Supercaps, 8 (2025) e202400466.

[7] P. Mahajan, S. Sardana, A. Mahajan, Ternary MXene/PANI/ZnO-based composite with a built-in p–n heterojunction for high-performance supercapacitor applications, Journal of Physics D: Applied Physics, 58 (2025) 045501.

[8] M. Gao, J. Shao, Y. Huang, X. Duan, S. Yang, F. Wang, F. Yu, Z. Liu, Y. Gao, P. Zhang, Porous MXene/transition metal dichalcogenide heterostructure anodes with accelerated charge transfer kinetics for high-energy and long-life sodium-ion hybrid capacitors, Chemical Engineering Journal, (2025) 168135.

[9] S. Shanmuganathi, T. Selvam, D. Dhinasekaran, A.K.M. Rajendran, B. Subramanian, A.R. Rajendran, Synergistic Ti_3_C_2_ MXene/MoS_2_ heterostructure: Unlocking ultra-fast, durable, and high-efficiency energy storage for next-generation supercapacitors, Applied Physics Letters, 126 (2025).

[10] K. Manoharan, M. Pumera, A band-aid-based MoSe_2_/Nb_2_C wearable supercapacitor for integrated ammonium-ion energy storage and real-time pressure monitoring, Journal of Materials Chemistry A, 13 (2025) 35533-35546.

[11] R. Sinha, P.S. Kiran, K.V. Kumar, N. Pandit, C. Satish, S. Keshri, A.K. Keshri, MXene/Biomass-derived activated carbon composite for supercapacitor applications, Carbon, 236 (2025) 120101.

[12] S.M. Varghese, R.S. Srihari, M.O. Anagha, R.B. Rakhi, Transition Metal Oxide–Decorated Titanium Carbide MXene Electrodes for High‐Energy Supercapacitors with an Expanded Potential Window, Batteries & Supercaps, 8 (2025) e202500304.

[13] Y. Luo, Y. Tian, Y. Tang, X. Yin, W. Que, 2D hierarchical nickel cobalt sulfides coupled with ultrathin titanium carbide (MXene) nanosheets for hybrid supercapacitors, Journal of Power Sources, 482 (2021) 228961.

[14] Y. Wang, J. Sun, X. Qian, Y. Zhang, L. Yu, R. Niu, H. Zhao, J. Zhu, 2D/2D heterostructures of nickel molybdate and MXene with strong coupled synergistic effect towards enhanced supercapacitor performance, Journal of Power Sources, 414 (2019) 540-546.

[15] S. Venkateshalu, J. Cherusseri, M. Karnan, K.S. Kumar, P. Kollu, M. Sathish, J. Thomas, S.K. Jeong, A.N. Grace, New method for the synthesis of 2D vanadium nitride (MXene) and its application as a supercapacitor electrode, ACS omega, 5 (2020) 17983-17992.

[16] W. Wang, D. Jiang, X. Chen, K. Xie, Y. Jiang, Y. Wang, A sandwich-like nano-micro LDH-MXene-LDH for high-performance supercapacitors, Applied surface science, 515 (2020) 145982.

[17] X. He, T. Bi, X. Zheng, W. Zhu, J. Jiang, Nickel cobalt sulfide nanoparticles grown on titanium carbide MXenes for high-performance supercapacitor, Electrochimica Acta, 332 (2020) 135514.

[18] Z. Pan, F. Cao, X. Hu, X. Ji, A facile method for synthesizing CuS decorated Ti_3_C_2_ MXene with enhanced performance for asymmetric supercapacitors, Journal of Materials Chemistry A, 7 (2019) 8984-8992.
